# Amino acids in sandal (*Santalum album L*) with special reference to *cis*-4-hydroxy-l-proline and *sym*. homospermidine

**DOI:** 10.1186/s40064-015-1303-1

**Published:** 2015-09-24

**Authors:** Ramadasan Kuttan, Beena Panikkar, Ponnamparambil Purushothaman Binitha

**Affiliations:** Amala Cancer Research Centre, Amala Nagar, Thrissur, Kerala 680555 India

**Keywords:** Sandal (*Santalum album*), Hydroxy prolines, *sym*. Homospermidine, Lachrymatory precursor, Biosynthetic pathways

## Abstract

Sandal (*Santalum album L*) contains several interesting amino acids and amines which are not seen in other plants. This includes *cis*-4-hydroxy-l-proline in free form in leaves, flowers and seeds while *trans*-4-hydroxy-l-proline in bound form. Traces of 3, 4 dehydroproline is also detected in sandal leaves. Biosynthesis of *cis*-4-hydroxy proline indicates that hydroxylation taken place at proline present in peptidyl form especially bound to glutamic acid and aspartic acid. Pyrrolizidine-2-carboxylic acid an interesting isatin positive heterocyclic compound is also present in sandal leaves. Sandal also contains *sym*. homospermidine which is not present in any other plants till today. Biosynthesis of *sym*. homospermidine goes by a unique pathway of putrescine oxidation, Schiff base formation, condensation and reduction. Moreover sandal leaves contain γ-glutamyl derivative of the lachrymatory precursor of onion, γ-glutamyl-S-propenyl cysteine superoxide. This review summarizes the studies on the amino acids in sandal.

## Background

Sandal is most probably indigenous to peninsular India, though some authorities feel that it was originally imported from Timor, Indonesia. About 65 % of the world’s sandal wood spreads across Karnataka, India. Sandal wood paste and oil has been extensively used as perfume from time immemorial. The wood contains an essential oil containing santalol and other terpenoid derivatives. Sandal is one of the most important plants used in many medicaments and perfumery shows the unusual presence of several interesting compounds. This includes *cis*-4-hydroxy-l-proline (Radhakrishnan and Giri 1954) (an optical isomer of *trans*-4-hydroxy-l-proline present in collagen) which is present in a free state, as well as a bound *trans*-4-hydroxy-l-proline (Kuttan and Radhakrishnan 1970) present as a soluble form and insoluble form. *sym*. Homospermidine (Kuttan et al. 1971) an analogue of polyamine spermidine as well as l-γ-glutamyl-S-propenyl cystein sulfoxide (Kuttan et al. 1974a, b) a stereo isomer of lachrymatory precursor present in onion is also reported to be present in sandal leaves. Moreover another isatin positive compound pyrrolizidine 2 carboxylic acid has also been isolated from sandal leaves. Possible presence of precursors of *cis*-4 hydroxy proline, such as 3, 4 dehydroproline and γ-hydroxy glutamic acid have also been checked in the leaves extract. This review is dedicated to Professor Dr. A. N. Radhakrishnan for his pioneering work in Biochemistry especially on amino acid metabolism.

### *cis*-4-Hydroxyproline in sandal

**Fig. 1:**
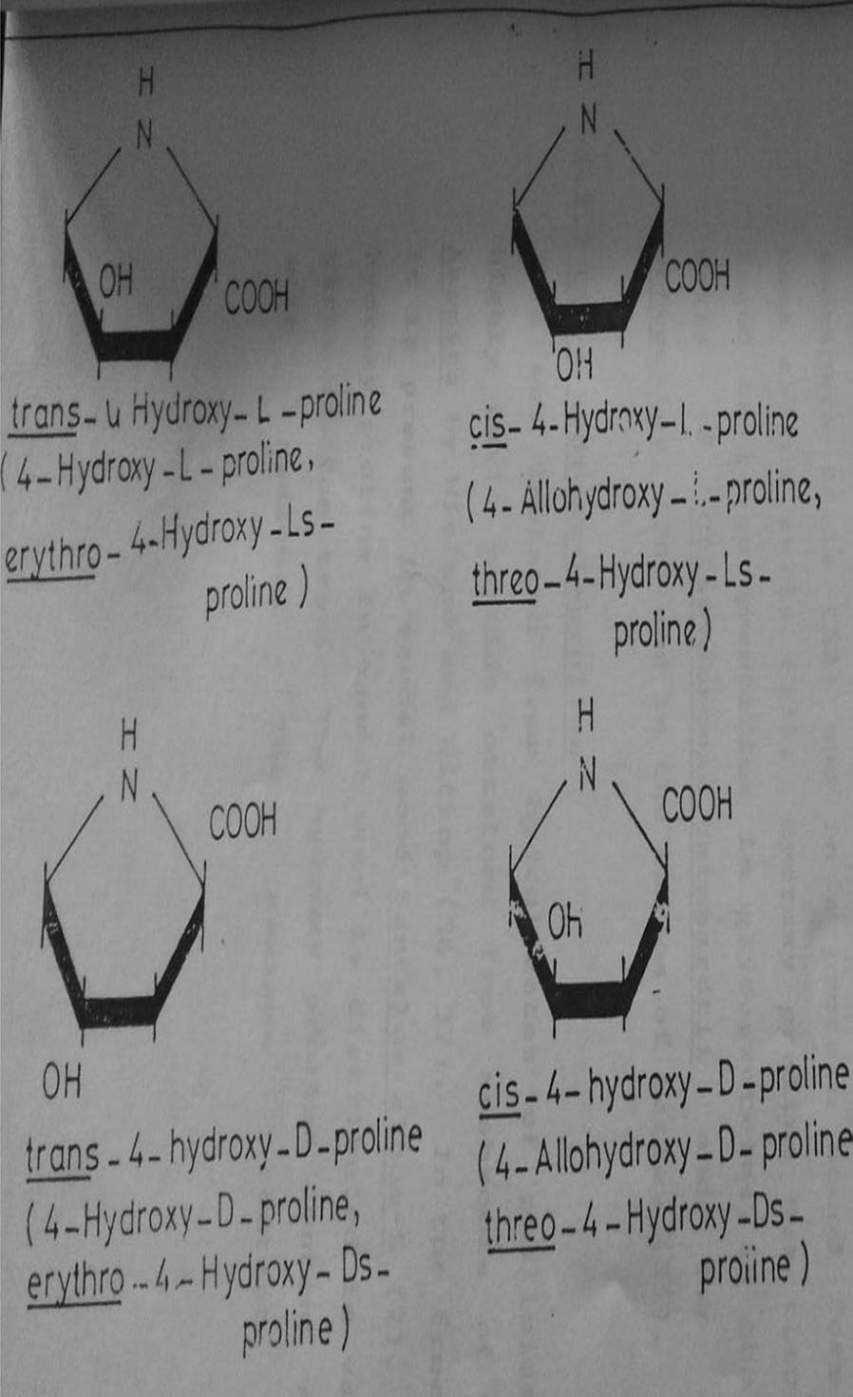
Structure of hydroxyproline

Hydroxy proline has two asymmetric carbons and hence can exist in four optically active forms (Fig. 1). *Trans*-l-hydroxyl proline was first solated from the hydrolyzates of gelatin in 1902 by Fischer (Fischer 1902) and named it as oxypyrolidine-carboxylic acid. *Trans* 4-hydroxy l proline is present in various collagens like proteins, reticulins, collastromin and elastoidin in dentine protein, horse radish peroxidase actinomycin X_0_B, sarcina lutea etc. It is also present in the cell wall of several plants (leaves, pericarp and roots) in a bound and is called Extensin as it is needed in the cell wall extension (Lamport 1965). *Cis*-4-hydroxyl-l-proline was isolated from hydrolyzates of phalloidin, a highly toxic peptide obtained from mushrooms of genus Amanita by Wieland and Witkop. *Cis*-4-hydroxy-l-proline is present in free state in *Santalum album* (Radhakrishnan and Giri 1954). It is also present in other Santalum species such as *Santalum yassi, Santalum austrocaledonicum, Santalum obtusifolium* (Kuttan et al. 1974a, b) as well as in *Osyrus arborea W,Thesium himalense Royal* and *Thesium wightianum wall* which are members of *Santalaceae* family. Excepting these species presence of *cis*-4-hydroxy proline is not reported in any other plants. In sandal, free hydroxyl proline is distributed in various parts of the tree such as in leaves and pericarp of fruits. The hydroxy proline content varied with season. In the vegetative phase young leaves had maximum content while in reproductive phase flowers and seeds had maximum content of *cis*-4-hydroxy-l-proline.

Biosynthesis of *cis*-4-hydroxy proline has been studied using radioactive precursors such as proline, ornithine, glutamic acid, acetate etc. (Kuttan and Radhakrishnan 1970) and it was found that maximum labeling into hydroxyl proline was obtained when proline was used. Ornithine labeling into hydroxyl proline followed the proline incorporation. It was proposed that *cis*-hydroxy proline was formed by the hydroxylation of proline. Possible biosynthetic pathways of *cis*-4-hydroxy-l-proline formation are discussed below.

A. Proline→*cis*-4-hydroxy proline.
B. Proline→3, 4 dehydroproline→*cis*-4-hydroxy-l-proline.
C. Proline→3, 4 dehydroproline→4-keto proline→*cis*-4-hydroxy-l-proline.
D. *cis*-4-hydroxy glutamate→*cis*-hydroxy-glutamic-γ semialdehyde→Δ′ pyrroline-4-hydroxy-2-carboxylate→*cis*-hydroxyl proline.
E. γ-Hydroxy ornithine→γ-hydroxy glutamic-γ-semialdehyde→Δ′-pyrroline-4-hydroxy-2-caroxylate→*cis*-4-hydroxy-l-proline.

### Role of 3, 4 dehydroproline in the biosynthesis of *cis*-4 hydroxy proline

**Fig. 2:**
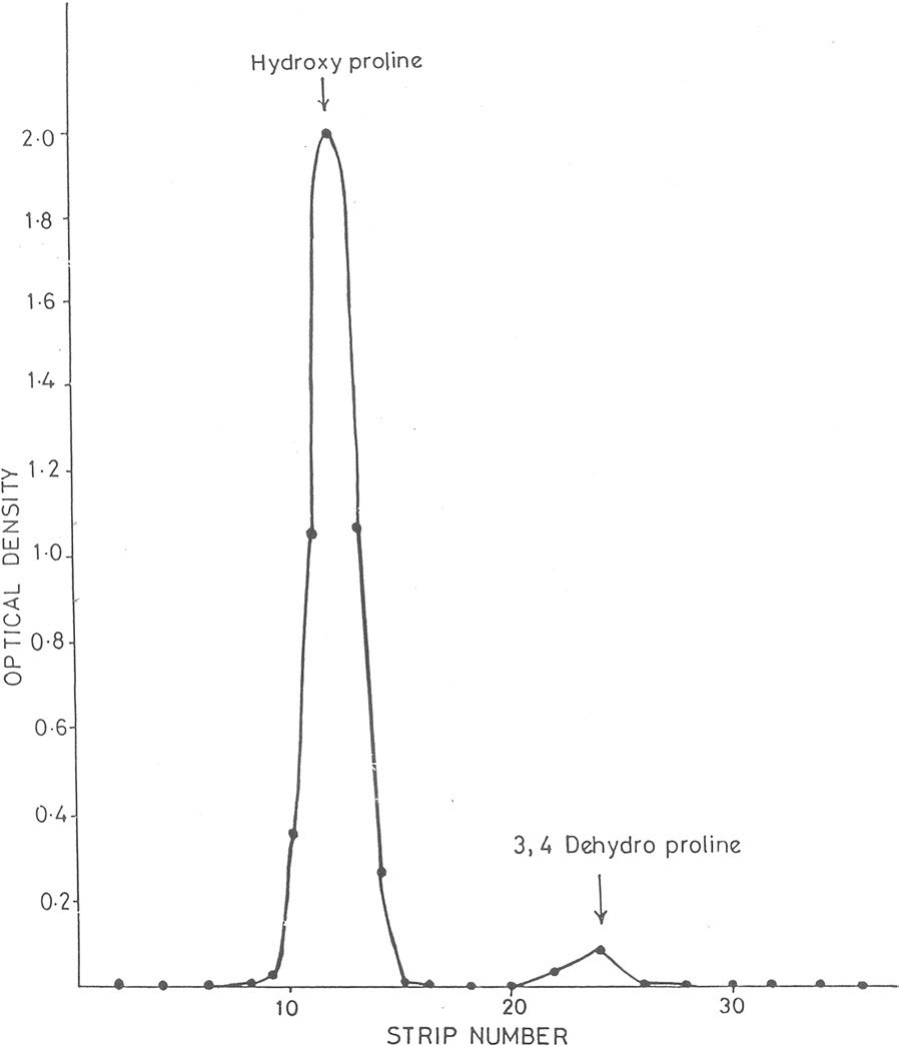
Demonstration of presence of 3, 4 dehydroproline in sandal leaf extract using paper chromatographic procedure

Our initial studies indicated that 3, 4 dehydroproline may be present in sandal leaves at very low concentrations. It was found that during the column separation 3, 4 dehydroproline which reacts with Ehrlich’s reagent was eluted much later than the hydroxyl proline. Fractions containing hydroxyproline and 3, 4 dehydroproline were pooled and concentrated and unidimensional chromatography using butanol-acetic acid–water (4:1:1) separated it from hydroxyl proline as a defined peak with an R_f_ value very similar to that of 3, 4 dehydroproline (Fig. 2).

Similarly after incorporation of radioactive proline into the sandal leaves radioactivity could be detected in the position similar to that of 3, 4 dehydroproline. The presence of 3, 4 dehydroproline in the sandal leaves was also found to be seasonal dependent. Upon administration of 3, 4 dehydroproline into sandal leaves this imino acid gets metabolized very fast. Hence 3, 4 dehydroproline in sandal leaves has only a very short half life. Presently it is not known whether 3, 4-dehydroproline is one of the intermediates in the biosynthetic pathway of *cis*-4-hydroxy-l-proline or a metabolic product produced from either proline or hydroxy proline.

### *trans*-4-hydroxy-l-proline in sandal

The sandal leaves also contained the natural isomer of hydroxyl proline i.e. *trans*-4-hydroxy-l-proline which has been shown to be present in a bound (associated with a protein) state (Kuttan and Radhakrishnan 1970). This constitutes nearly 4 % of the total hydroxyl proline present in sandal leaves.

Hydroxy proline in bound state was mainly associated with a wall and soluble fraction together constituting nearly 86 % of total bound hydroxyl proline in leaves. Bound hydroxy proline is present only in small amounts in the soluble fraction but is mostly associated with “wall” fraction.

Various extraction procedures showed the heterogeneity of bound proline containing components in sandal. Hot 5 % (W/V trichloroacetic acid extracts about 25 % of hydroxyl proline and M NaOH extracted an additional 25 %. All these fractions had varying ratios of proline:hydroxyl proline, hydroxy proline:sugar and hydroxyl proline:protein.

Using radioactive labeling techniques it was shown that hydroxyl proline in soluble fraction and wall fractions were synthesized from proline. Hydroxylation of proline takes place in the peptide form during ribosomal elongation of the chain. ATP and Mg^++^ are needed for elongation and ascorbate and Fe^++^ for hydroxylation. Enzyme responsible is prolyl hydroxylase. Pulse labeling studies indicated that soluble fraction is not a precursor of wall fraction.

Bound hydroxyl proline content in the soluble and in the cell wall fractions was determined in younger and in progressively older leaves. It was seen that trichloroacetic acid soluble bound hydroxyl proline decreased from buds to older leaves. The soluble hydroxyl proline containing protein of sandal leaves has been purified to homogeneity (Mani and Radhakrishnan 1974), by Sephadex G-50 column and further absorption on alumina gel and the protein was purified 17-fold with a recovery of 23 %.

### Infiltration studies using radioactive proline

When the ^14^C-proline was infiltrated into the sandal leaves the radioactivity was found to be associated not only with proline but also with hydroxy proline. The incorporation of proline into the hydroxyproline at various intervals of time such as 2, 6, 24 h were determined. The incorporation of proline to hydroxy proline was initially was low in the beginning reaching a maximum at 6 h and further reduced at 24 h. The radioactivity in hydroxyproline to proline ratio was initially was very low and became almost one at 24 h.

### Identification of an intermediate in the biosynthesis of *cis* 4 hydroxy proline

**Fig. 3:**
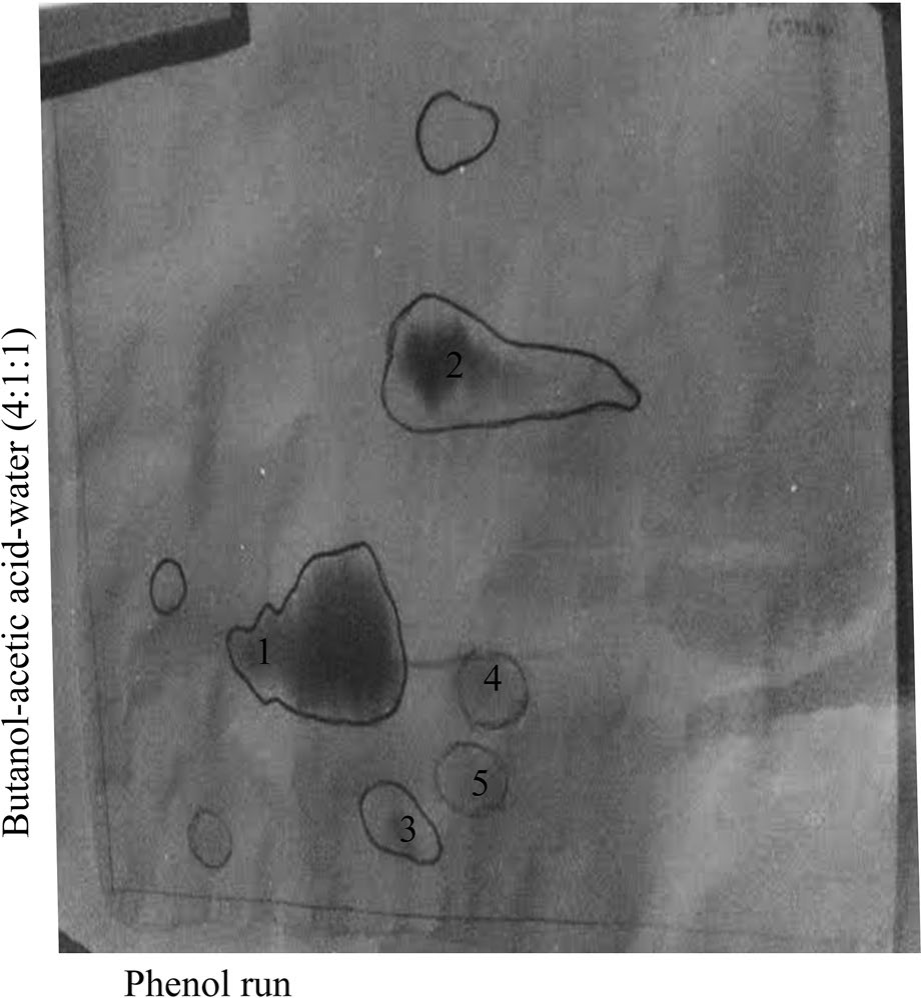
Two dimensional paper chromatography of Ehrlich positive material. *1* *cis*-4-hydroxy proline. *2* Proline. *3*, *4*, *5* Ehrlich positive material

Earlier work by Radhakrishnan indicated that the sandal leaf extract contained a material that reacted with Ehrlich reagent (unpublished). This material remained in the origin during paper chromatography using butanol-acetic acid–water as solvent systems. When 0.75 N HCl elutes of sandal extracts were analysed by paper chromatography there was documented evidence of at least two Ehrlich positive materials which appeared below hydroxyl proline. Large scale isolation of Ehrlich positive material was attempted using Dowex column chromatography and further purification of this material using paper chromatography. The purified material reacted faintly with ninhydrin. Two dimensional paper chromatography of the Ehrlich positive material indicated mobility similar to hydroxyl proline in phenol run and below hydroxyl proline in butanol run (Fig. 3). The R_f_ values of Ehrlich positive material in butanol-acetic acid–water (4:1:1) system was 0.25 and in phenol–KCl:HCl system was 0.57. The partially purified material was found to be highly unstable and it was converted to hydroxyl proline. Stereo configuration of hydroxy proline produced by hydrolysis was found to be *cis*-4-hydroxy-l-proline using amino acid analyser in which *cis* and *trans* hydroxyproline separated.

The nature of the Ehrlich positive material is not known. The following evidences indicated that the Ehrlich positive material is not an artifact of isolation but is involved in the biosynthesis of *cis*-4-hydroxy-l-proline (a) Ehrlich positive material as well as material in the origin increased after cold proline incorporation (b) when infiltration with ^14^C proline label could be seen in the origin which was converted to the Ehrlich positive material and to *cis*-hydroxyl proline (c) during the isolation of the material in the origin loosely bound hydroxyproline could be seen in the chromatogram (d) after proline incorporation ^14^C hydroxyproline increased in the material present in the origin (e). Ehrlich positive material produced *cis*-4-hydroxy-l-proline after hydrolysis. The other amino acid that is produced after hydrolysis of the Ehrlich positive material was found to be glutamic acid and aspartic acid.

**Fig. 4:**
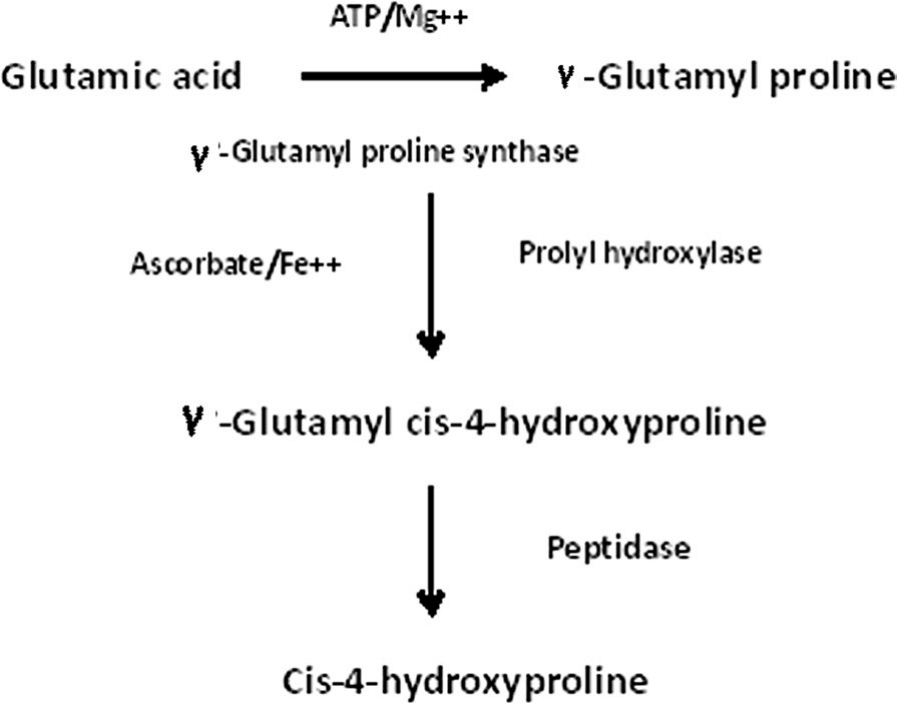
Biosynthesis of *cis*-4-hydroxyproline

These evidences indicate that the *cis*-hydroxy proline synthesis in the sandal leaves may be taking place in the peptide chain (Fig. 4). Hydroxylation reaction may be taking place on peptide rich in acidic amino acids such as glutamic acid and aspartic acid. It is tempting to speculate that γ-glutamyl proline or β-aspartyl proline are the possible substrates for the hydroxylase. The molecular weight of the substrate is not known at present. γ-Glutamyl compounds are reported in sandal as well as in other plants as nitrogen storing material. However their role in producing hydroxy proline needs to be further studied.

The inability of other precursors such as glutamic acid and ornithine to produce a significant increase in *cis*-hydroxyl proline levels in sandal leaves indicates that hydroxyl prolines in the plant is not produced by cyclisation reactions of these five carbon amino acids. Similarly since proline did not directly produce *cis*-4-hydroxy-l-proline even when its concentration was very high indicates that proline incorporation into *cis*-4-hydroxy-l-proline may possibly takes place by indirect methods such as peptide synthesis, hydroxylation and cleavage. Other precursors seen as 3, 4, dehydroproline or 4-keto proline may not be involved in this conversion.

### Identification of two new amino acids from sandal leaves

200 g of dried sandal leaves powder was extracted with one liter of 75 % methanol and kept overnight. Supernatant was filtered and to the residue 500 ml of 75 % methanol was again added and kept overnight. It was filtered and the residue was extracted again using 500 ml 75 % methanol. All the supernatants were pooled together and concentrated in a boiling water bath to 100 ml. There after, 200 ml of chloroform was added and aqueous layer was collected by centrifugation. The pH of the aqueous layer was adjusted to 1.0 and kept overnight. Precipitate formed was removed next day by centrifugation. The supernatant was passed through a Dowex (50 × 8) H^+^ column (110 cm × 2 cm). The column was first washed with 500 ml water which was collected as 50 ml fractions (10 numbers). Thereafter the column was washed with 500 ml 0.75 N HCl. 10 ml fractions were collected (50 numbers). Further the column was washed with 500 ml 1.5 N HCl which was collected and stored. 10 µl of the alternate fractions collected with 0.75 N HCl were spotted on Whatman No. 1 paper and unidimensional paper chromatography was done with phenol–KCl–HCl (50:7) buffer system and butanol-acetic acid–water (4:1:1) as solvent system using hydroxyl proline and proline as the standards. The chromatograms were sprayed with 0.4 % ninhydrin in acetone containing 2 % collidine. Another set of chromatograms were sprayed with isatin (0.4 %) in acetone and heated at 70 °C for 10 min. The fractions containing the new amino acids appeared in fraction nos. 16–50. The fractions were pooled and dried in the boiling water bath (Fraction 1 and Fraction II).

### Purification of aminoacid in fraction I

The fraction I was made up to 2 ml and streaked on Whatman No. 1 paper and chromatographed in phenol–KCl–HCl buffer pH 2.0. The new amino acid present in this fraction did not have any mobility in this system and hence the material in the origin was cut and eluted with water. The elutes were pooled and concentrated to dryness using a lyophilizer. The lyophilized amino acids are made up to 2 ml and were passed through a Dowex (50 × 8H^+^) column (30 cm × 1.5 cm). After washing with water the new amino acid was eluted with 1 N ammonia. The ammonia elute was evaporated to dryness using a lyophilizer Yield = 100 mg.

### Purification of amino acid in Fraction II

The fraction 16–50 from 0.75 N HCl elute was concentrated to dryness in a boiling water bath and made up to 2 ml. This material was streaked on a Whatman No. 1 paper and chromatographed using butanol-acetic acid–water (4:1:1) with hydroxyl proline and proline as standards. The new amino acid appeared as an isatin positive material above proline. This material was cut out and eluted with water. The pooled elute was concentrated by lyophilization and made up to 2 ml. The fraction was further passed through a Dowex (50 × 8 H^+^) column (30 cm × 1.5 cm) and the column was washed with water (100 ml) and further eluted with 150 ml 1 N ammonia. The ammonia elute was lyophilized to dryness yield = 126 mg.

**Fig. 5:**
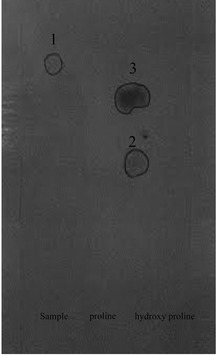
Paper chromatogram of isatin positive material in phenol–KCl: HCl Buffer (*1*) Isatin positive material, (*2*) proline, (*3*) hydroxyproline

Paper chromatography of the isolated material is shown in the (Fig. 5).

### Nature of the amino acids

R_f_ of the two amino acids as compared with that of proline, hydroxyl proline and γ-hydroxy glutamate in two different solvent systems are given in (Table 1). The new amino acid in fraction 1 was found to have R_f_ value 0.01 in phenol–KCl–HCl buffer and 0.11 in butanol-acetic acid–water system (4:1:1). On the other hand the new acid present in fraction II was found to have an R_f_ value (0.65) in phenol–KCL–HCl buffer and 0.78 in butanol-acetic acid–water (4:1:1). The new amino acid in fraction II had a different mobility than phenyl alanine, valine, leucine which also appeared in the similar position.

**Table 1:** Chromatographic mobility of isolated amino acids

Sample	R_f_ values	
	Butanol-acetic acid–water system	Phenol–KCl:HCl system
Fraction 1	0.11	0.01
Fraction 11	0.78	0.65
Hydroxy proline	0.51	0.52
Proline	0.68	0.66
γ-Hydroxy glutamate	0.32	0.19

Both the amino acids were found to give positive reaction to ninhydrin (purple colour). Aminoacid in Fraction II was also found to give blue colour similar to proline using isatin reagent with almost same sensitivity. However unlike proline which gives yellow color with ninhydrin this material produced purple color.

### The purity of the isolated material

The material isolated on the sandal leaves was not completely pure. Aspartic acid was found to be the major contaminating amino acid in fraction I. Fraction II gave two ninhydrin positive materials of which the lower spot was found to be isatin positive. Because of the closely related mobility some amino acids such as tryptophan, phenyl alanine or tyrosine could be a possible contaminant in this fraction.

### Identification of isatin positive material

**Fig. 6:**
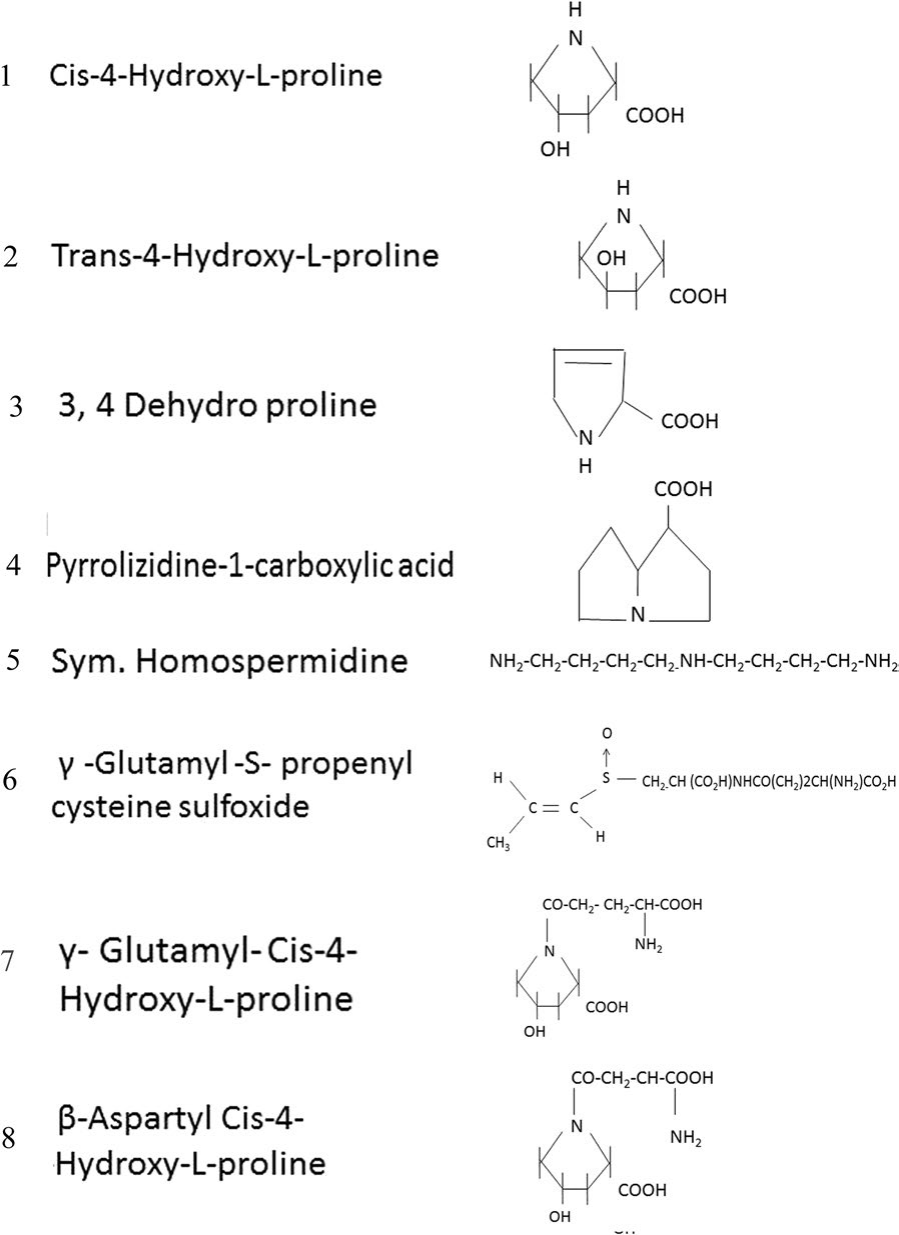
List of amino acids and amines isolated from sandal leaves

The identification of the amino acids was done by Dr. T.F. Spande, National Institute of Diabetes and Digestive and kidney diseased, Bethesda, Maryland, U.S.A. The isatin positive material (Fraction II) was not found to be acelyted by acetic anhydride (2 h at room temperature) suggesting a tertiary amine. It was converted with CH_3_OH–BCL_3_ to a methyl ester (FTIR 1751 CM-1 Bohlmann) which chromatographed on GC–MS giving M/Z 169 (20, 154 (10), 141^15^, 138 (15), 110 (10), 108 (15), 83 (70), 82 (70) etc. A mass spectrum very similar to that reported for a diastereomeric ethyl ester isolated from an orchid. A 2D proton cosy spectrum established connectivities and indicated the above (or enantiomeric) structure unambiguously. This was further confirmed by ^13^C NMR and a proton carbon correlation spectrum (500 MHz). A sample was crystallized from CH_3_OH to EtOAc twice and had M.P. 213–5^o^ (lit 215–6^o^) and a rotation of (α) D = 30.5o (H_2_O). These evidences indicated the structure of isatin-positive material is pyrrolizidine-1-carboxylic acid (Fig. 6).

The ninhydrin positive material present in the fraction I was isolated because of its close mobility in paper chromatography with γ-hydroxy glutamate. However further investigation by paper chromatography proved that it may not be γ-hydroxy glutamate.

### *sym*. Homospermidine: a new polyamine from sandal leaves

#### Isolation

**Fig. 7:**
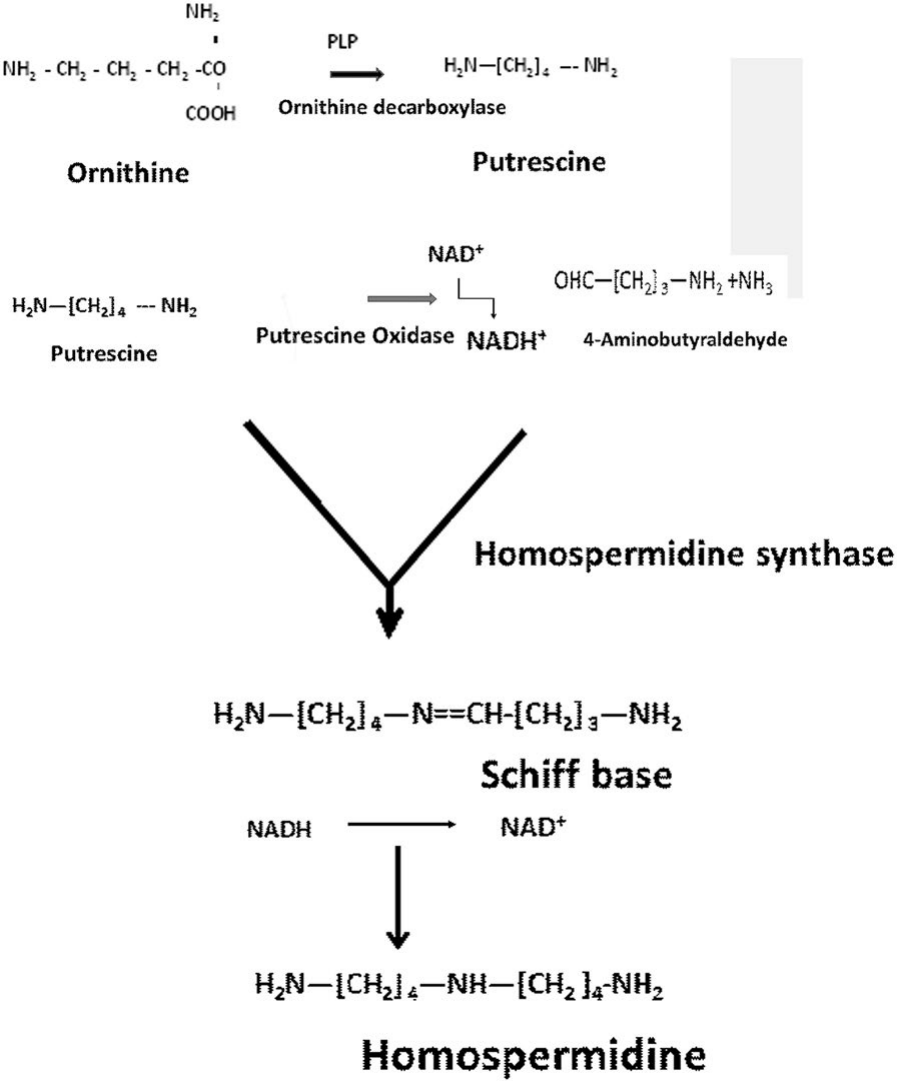
Biosynthesis of *sym*. homospermidine

Two dimensional chromatography of an alcoholic extract of sandal leaves showed prominent ninhydrin positive malene near the origin in butanol acetic acid water (4:1:1). During Dowex 50 × 8H^+^ column chromatography this material was not eluted with 1 N ammonium hydroxide in which most of the amino acids eluted. When the column was further eluted a stronger base piperidine (1 M) this ninhydrin positive material was found to be eluted as a single spot. Operation of this procedure using longer column led to the purification of this compound which was further characterized by NMR IR spectrum and mass spectrum analysis as *sym*. homospermidine, a new polyamine, not reported earlier from any other system. (yield was 1.5 g). Structure of the *sym*. homospermidine was seen in Fig. 7. Moreover synthetic homospermide hydrochloride was prepared which superimposed on that of natural homospermidine. Chromatographic behavior of both was identical.

Quantitation in the leaves was done using small Dowex 50 × 8H^+^ column. After loading a known quantity of the extracts column was washed with 1 N ammonium hydroxide (10 bed volume) and further with 10 bed volume of peperidine (1 M). Piperidine eluent was concentrated to dryness and quantitated by Rosen’s procedure (Rosen 1957) using leucine was the standard. It was found that homospermidine was found in the leaves at a range of 0.5–1.5 % of the weight of the dried leaves.

#### Biosynthesis of *sym*. homospermidine

Biosynthesis of *sym*. homospermidine was studied using radioactive migration studies. Both arginine and ornithine are good precursors of homospermidine. Arginine appears to be more effective precursor than ornithine. After infiltration of the leaves with [^14^C] arginine and [^14^C] ornithine, the ethanolic extracts were subjected to two dimensional paper chromatography and radioautography. With arginine as the precursor the label was found in homospermidine, agmatine, putrescine, ornithine, proline and γ-aminobutyrate. With ornithine as the precursor the label was found in homospermidine, glutamate, proline, citrulline putrescine, γ-aminobutyrate, aspartate and arginine.

#### Biosynthetic pathway for homospermidine in sandal leaves

In view of the symmetrical nature of the homospermidine molecule it was further considered that both half of the molecule was derived from putrescine. The biosynthesis may then involve a Schiff-base formation between putrescine and γ-aminobuteraldehyde, a metabolite of putrescine. Further reduction of the Schiff base would yield homospermidine. Such a mechanism is supported by the observed incorporation of ^3^H from the medium into homospermidine, suggesting a nucleotide-dependent reduction. Also in model systems containing γ-aminobuteraldyhyde and putrescine followed by reduction with NaBH4 or Pt/H2, the formation of homospermidine was demonstrated. On the basis of these results a scheme for the biosynthesis of homospermidine in sandal is presented (Fig. 7).

An enzyme catalysing the synthesis of *sym*. homospermidine from putrescine and NAD^+^ with concomitant liberation of NH3 was purified 100-fold from *Lathyrus sativus* (grass pea) seedlings by affinity chromatography on Blue Sepharose. This thiol enzyme had an apparent mol wt. of 75,000 and exhibited Michaelis–Menten kinetics with Km 3.0 Mm for putrescine. The same enzyme activity could also be demonstrated in the crude extracts of sandal (*Santalum album*) leaves, but with a specific activity 15-fold greater than that in *L. sativus* seedlings (Srivenugopal and Adiga 1980). Tait (1979) purified an enzyme when synthesize of homospermidine from Rhodopseudomonus viridis. Biosynthetic pathway of homospermidine was similar to that is same as sandal as reported.

Polyamines, putrasines, spermidine and speemine are present in the animal tissues and biological fluids. While putrascine is ubiquitously present in plants higher polyamines are seldom present. In this respect discovery of homospermidine in sandal leaves is highly interesting. Homospermedine in sandal leaves is detected in alcohol and H_2_O extract and is present in an unconjugated form. Till today no other species of animals, plants, microorganisms have been reported to have *sym*. homospermidine.

### γ-l-Glutamyl-S-(*trans*-1-propenyl)-l-cysteine sulfoxide

During the routine analysis of the amino acids in sandal using two dimensional chromatography another unknown ninhydrin positive was observed. This compound was an acidic amino acid which is in the amino acid analyser emerged 15 min earlier than trans-4 hydroxyproline. Acidity of this amino acid was exploited in isolating this amino acid in pure form. Chemical hydrolysis of GPCS from sandal leaves yielded glutamic acid and cysteine. Milder acid hydrolysis liberated glutamic acid and indicated the presence of a γ-glutamyl linkage in the peptide, a peptide in which glutamic acid was also N-terminal as indicated by the DNP assay. Acid and enzymatic hydrolysis combined with proton magnetic resonance, circular dichorism and IR spectrometry established the structure of the unknown amino acid and γ-glutamyl S-propenyl cysteine, sulfoxide (GPCS).

**Fig. 8:**
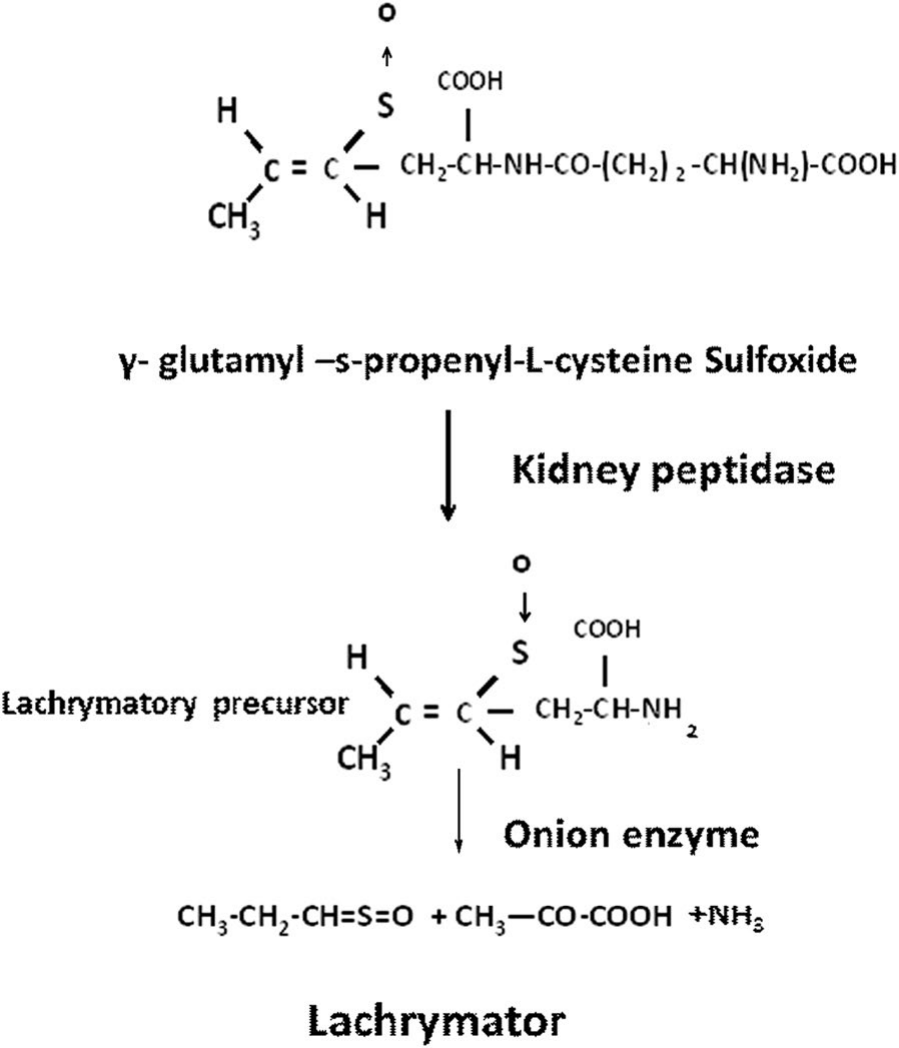
Production of lachrymatory factor from γ-glutamyl-s-propenyl-l-cysteine Sulfoxide

The sulfoxide diasterioisomer of GPCS had previously been isolated from onion (*Allium cepa*) by Virtanen and Matikkala (Virtanen and Matikkala 1961), who showed that cleavage of the glutamyl peptide bond with a beef kidney preparation, yielded the so called lachrymatory precursor, also found in the onion. When lachrymatory precursor was exposed to an enzyme released from crushed onion, pyruvate, ammonia and the onion lachrymatory factor are formed (Fig. 8).

#### Quantitation in leaves

The leaves from young plants contained only traces while much larger amounts were found in older plants. The amino acid analyzer permitted a quantitative estimation of the peptide in the plant leaves from which the peptide was isolated. The peptide constituted nearly 0.5 % of the dried leaves. The γ-l-glutamyl peptide of s-(1-propenyl)-l-cysteine sulfoxide is the principal γ-glutamyl peptide of onion (*Allium cepa*). Fresh onions contain nearly 0.2 % by weight while dehydrated onions contained 0.15 % of GPCS. Lesser amounts of γ-glutamyl-S-(2-carboxylpropyl) cysteine and S-methyl cysteine γ-glutamyl peptides of S-alkylated cysteine are also found in garlic (*Allium sativum*) and chives (*Allium shoenoprasum*). Cysteine derivatives e.g. S-methyl, S-propyl-and S-(1-propyl-cysteine, occur in onion as both the thioester and sulfoxide form (Sugii et al. 1963). S-Allyl cysteine as the thioester in onion and the sulfoxide in garlic but the γ-glutamyl peptides of these cysteine derivatives are rarely found in the oxidized state.

The proton magnetic spectrum of GPCS from sandal provides unambiguous evidence that the alkyl group attached to cysteine is an S (1 propenyl) group with *trans* configuration. Most of the optically active sulfoxides which have been isolated from natural sources and whose sulfoxide configurations have been determined are of the S configuration (Lucas and Levenbook 1966). The single exception seems to be the class of isothiocyanate sulfoxides and suforaphene found in mustard oil. The various γ-glutamyl-peptides including GPCS disappear from the bulbs of sprouting onion and garlic and these may therefore function as a nitrogen reserves. No other role has apparently been proposed for these unusual peptides. The occurrence of GPCS in a higher plants unrelated to the Allium genus is very rare as is the finding that the peptide concentration is greater in mature plants than in young plants.

### Role of hydroxyproline and *sym*. homospermidine in sandal

*trans*-4-hydroxyproline is a major amino acid in collagen in which it is present nearly 10 % of the total amino acids. It produces the tensile strength of the collagen. In plants *trans* 4-hydroxyproline is present as a part of the cell wall protein and is helpful in the cell wall extension. No specific role of *cis*-4-hydroxyproline has been attributed in plants or animal kingdom. Incorporation of *cis*-4-hydroxy proline by prolyl S-RNA has been reported and collagen incorporated with *cis*-4-hydroxyproline has been reported to be less stable and hence used in reducing fibrosis. However no physiological role of *cis*-4-hydroxyproline has been reported in sandal which in fact has *trans*-4-hydroxyproline in bound form.

Similarly there is no role of *sym*. homospermidine has been reported in plants. Spermine and spermidine present in animal kingdom has been reported to have a stabilizing effect on DNA during replication. Both these polyamines are not present in plants. Presence of *sym*. homospermidine in sandal is hence an isolated incidence. However no physiological role can be attributed expecting its catonic role. Sandal is a root parasite and hence the presence of *sym*. homospermidine may have a role as a catonic during its early stages of growth.

At present no specific use of these compounds has been reported in any disease condition including cancer excepting in reducing fibrosis as discussed earlier.

## Conclusion

Sandal is a store house of several interesting amino acids and amines. This includes *cis*-4-hydroxy-l-proline present in the free form in leaves and fruit pericarp. Leaves also contain *trans*-4-hydroxy proline in bound form both as wall fraction and as a soluble form. Biosynthesis of *cis*-4-hydroxy proline indicate that hydroxylation takes place at the γ-glutamyl proline and β-aspartyl proline, as γ-glutamyl and β-aspartyl hydroxyl proline could be detected as Ehrlich’s positive material in sandal extract. Limited evidence indicate that sandal leaves contain 3, 4 hydroxy proline in small quantities while γ-hydroxy glutamate or γ-hydroxy ornithine were absent. Sandal leaves also contains another Isatin positive compound pyrrolizidine-1-carboxylate. However it may not have any relation with the *cis*-hydroxy proline synthesis. Sandal leaves also contain nearly 1 % *sym*. homospermidine which was isolated using ion exchange column and structure determination is done by physicochemical method. *sym*. Homospermidine is rarely detected in nature and biosynthesis involves a pathway unknown in mammalian system. This involves putrescine oxidation to γ-aminobutyraldehyde and further Schiff base condensation with another molecule of putrescine and reduction. Interestingly enzyme catalyzing the homospermidine synthesis is also present in Lathyrus seedlings but compared to sandal leaves activity was low. Another compound which is present in sandal leaves is diasterioisomer of γ-glutamyl propenyl cysteine sulfoxide present in onion. Isolation of this compound was effected by ion exchange column and characterization of this compound was done by physiochemical method. List of amino acids and amines isolated from sandal are given in Fig. 6 and Table 2.

**Table 2:** List of amino acids and amines isolated from sandal

Amino acid (reproductive phase)	Source	Quantity
*Cis*-hydroxyl proline (free)	Leaves	1.2 %
	Pericarp	10 %
	Flowers	3.28 %
*Trans*-4-hydroxy proline (bound)	Leaves	0.04 %
3, 4 dehydroproline		Traces
γ-Glutamyl *cis*-4-hydroxy proline		Not estimated
β-Aspartyl *cis*-4-hydroxy proline		Not estimated
Pyrrolizidine 1-carboxylate		0.07 %
*sym*. Homospermidine		0.5–1 %
γ-Glutamyl-S-propenyl cysteine sulfoxide		0.5 %

Values are expressed as grams/100 g of dried material
